# Chloroplast Pan-Genomes and Comparative Transcriptomics Reveal Genetic Variation and Temperature Adaptation in the Cucumber

**DOI:** 10.3390/ijms24108943

**Published:** 2023-05-18

**Authors:** Lei Xia, Han Wang, Xiaokun Zhao, Hesbon Ochieng Obel, Xiaqing Yu, Qunfeng Lou, Jinfeng Chen, Chunyan Cheng

**Affiliations:** National Key Laboratory of Crop Genetics & Germplasm Enhancement and Utilization, College of Horticulture, Nanjing Agricultural University, Nanjing 210095, China; 2019204026@njau.edu.cn (L.X.);

**Keywords:** chloroplast pan-genome, cucumber, genetic variation, RNA editing efficiency, temperature adversity

## Abstract

Although whole genome sequencing, genetic variation mapping, and pan-genome studies have been done on a large group of cucumber nuclear genomes, organelle genome information is largely unclear. As an important component of the organelle genome, the chloroplast genome is highly conserved, which makes it a useful tool for studying plant phylogeny, crop domestication, and species adaptation. Here, we have constructed the first cucumber chloroplast pan-genome based on 121 cucumber germplasms, and investigated the genetic variations of the cucumber chloroplast genome through comparative genomic, phylogenetic, haplotype, and population genetic structure analysis. Meanwhile, we explored the changes in expression of cucumber chloroplast genes under high- and low-temperature stimulation via transcriptome analysis. As a result, a total of 50 complete chloroplast genomes were successfully assembled from 121 cucumber resequencing data, ranging in size from 156,616–157,641 bp. The 50 cucumber chloroplast genomes have typical quadripartite structures, consisting of a large single copy (LSC, 86,339–86,883 bp), a small single copy (SSC, 18,069–18,363 bp), and two inverted repeats (IRs, 25,166–25,797 bp). Comparative genomic, haplotype, and population genetic structure results showed that there is more genetic variation in Indian ecotype cucumbers compared to other cucumber cultivars, which means that many genetic resources remain to be explored in Indian ecotype cucumbers. Phylogenetic analysis showed that the 50 cucumber germplasms could be classified into 3 types: East Asian, Eurasian + Indian, and Xishuangbanna + Indian. The transcriptomic analysis showed that *matK* were significantly up-regulated under high- and low-temperature stresses, further demonstrating that cucumber chloroplasts respond to temperature adversity by regulating lipid metabolism and ribosome metabolism. Further, *accD* has higher editing efficiency under high-temperature stress, which may contribute to the heat tolerance. These studies provide useful insight into genetic variation in the chloroplast genome, and established the foundation for exploring the mechanisms of temperature-stimulated chloroplast adaptation.

## 1. Introduction

The cucumber (*Cucumis sativus* L.) originated in India, and is one of the major vegetable crops [1]. As the first horticultural crop whose whole genome has been sequenced, cucumber has been studied extensively in several areas, including sexual differentiation and vascular bundle formation [2]. *Cucumis sativus* var. hardwickii, whose plant is diminutive and used as an herbal cure, has been identified as the wild ancestor of cucumber, according to earlier research [3]. Wild cucumber has been transformed via crop domestication into delectable vegetables that are widely cultivated worldwide [4]. Depending on geographical location, cucumber germplasm resources can be divided into four groups: the Indian, East Asian, Eurasian, and Xishuangbanna groups [3,5,6]. To better understand the evolutionary mechanisms of phenotypic diversity in cucumber, a huge number of genome sequencing projects have been undertaken, such as the resequencing of 115 cucumber core germplasms [3] and the graph-based pan-genome [4] of cucumber. Although the cucumber genome has been extensively studied, very little research has focused on the organelle genome. Until now, only 13 cucumber organelle genomes have been reported, including 12 chloroplast genomes [7,8,9,10,11,12,13] and 1 mitochondrial genome [14]. Performing a chloroplast pan-genome study can deepen the understanding of cucumber genetics.

Chloroplasts are key organelles in plants that are involved in important biological processes such as photosynthesis and plant immunity [15]. There are three sets of genetic material in plant cells: the nuclear genome, the mitochondrial genome, and the chloroplast genome [16]. In contrast to the nuclear genome, the chloroplast and mitochondrial genomes are typically inherited maternally [17]. The chloroplast genome is a typical circular DNA module that ranges in size from 115 to 180 kb, multiple copies of which exist in the cell [18]. Chloroplast genomes have a typical quadripartite circular structure, including a large single copy (LSC) region, a small single copy (SSC) region and two inverted repeats (IRs) regions [19]. Based on the low mutation rate feature, chloroplast genomes provide valid information about plastid genome evolution as well as plant phylogeny [20,21]. In recent years, chloroplast genome sequencing has been completed in species such as *Ginger* [22], *Asteraceae* [23], and *Brassicaceae* [24], and different hotspots have been identified that can be used for phylogenetic analysis [25,26]. Furthermore, chloroplast genome sequences have been used for population genetics analyses. For example, 412 rice chloroplast and mitochondrial genomic analyses revealed that indica and japonica rice underwent different domestication processes [27]. With the continuous development of sequencing technology and improvement of chloroplast genome assembly technology, chloroplast pan-genome analysis has been carried out in several species such as oilseed rape [28], Japanese apricot [29], sweet potato [30], and pecan [31], which have explained their origin, evolutionary, phylogeography, and genetic diversity. Meanwhile, performing chloroplast pan-genome studies and analyzing the overall polymorphism of chloroplast genome sequences are important to explore candidate loci for genetically related agronomic traits in chloroplasts. For example, cold tolerance in cucumber is regulated by the F1FO-ATP synthase (CF1FO-ATPase) beta-subunit gene (*atpB*) [32].

In this study, the chloroplast pan-genome was constructed to explore the genetic diversity of 121 re-sequenced cucumber germplasm resources. Comparative transcriptomics clarifies how cucumber plastid genes respond to temperature stress. These results provide a basis for the study of chloroplast pan-genomes, genetic diversity, and adaptation to temperature stress in cucumbers.

## 2. Results

### 2.1. General Characteristics of 50 Cucumber Chloroplast Genome

**Figure 1:**
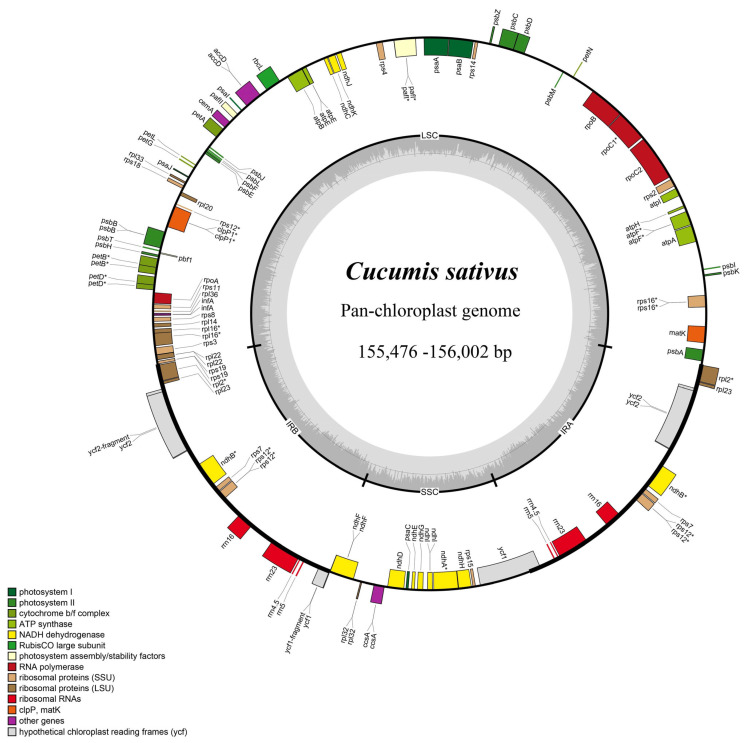
Pan-chloroplast genome map of *Cucumis sativus*. * Represents a gene containing an intron.

A total of 50 complete chloroplast genomes were successfully assembled and annotated from 121 samples. These chloroplast genomes range in length from 156,616–157,641 bp, but have a common 37% GC content (Table S1, Figure 1). These chloroplast genomes have a typical quadripartite molecular structure, including LSC (86,339–86,883 bp), SSC (18,069–18,363 bp), and two IRs (25,166–25,797 bp). Comparative genome analysis with the reference cucumber variety “GY14” revealed good co-linearity of our assembled chloroplast genome (Figure S1), demonstrating that the assembly of all chloroplast genomes was accurate. The annotation information revealed that the chloroplast genome has 85 common protein-coding genes, 37 common tRNA genes, and 8 common rRNA genes (Table S2), where *ndhB*, *rps7*, *rps12*, *rpl2*, *rpl23*, and *ycf2* have two copies. Among the protein-coding genes, 12 genes contained introns, namely *rps16*, *atpF*, *rpoC1*, *ycf3*, *rps18*, *rps12*, *rpl2*, two copies of *ycf2*, two copies of *ndhB*, and *ndhA*.

Based on chloroplast genome size differences, we classified these 50 cucumber chloroplast genomes into 23 categories (Table S3). In the East Asian-type cucumbers, 17 germplasm resources were classified into 4 categories: Huang Gua, Qiu Huang Gua, Liao Tong Mi Ci, and Jia Huang Gua. In the Eurasian-type cucumbers, the 11 germplasm resources were classified into 6 categories: Altaisky Ranny, SC 50, 8181, EC1, Ames 1208, and SC 53-B (6). In the Xishuangbanna-type cucumbers, 8 germplasm resources were classified into 4 categories: SWCC1, SWCC6, Man Wa Yuan Zong Huang Di Huang Gua, and SWCC8. In the Indian-type cucumbers, 14 germplasm resources were classified into 9 categories: CUS 155, CUS 233, USM 414, Hw 2, 8288, 11621, and Hw 3. Meanwhile, we found that the chloroplast genome of Indian-type cucumbers is rich in genetic variation compared to the other three types, which demonstrates the loss of genetic variation in cucumbers during domestication by artificial selection.

### 2.2. Comparative Analysis of Chloroplast Genome

**Figure 2:**
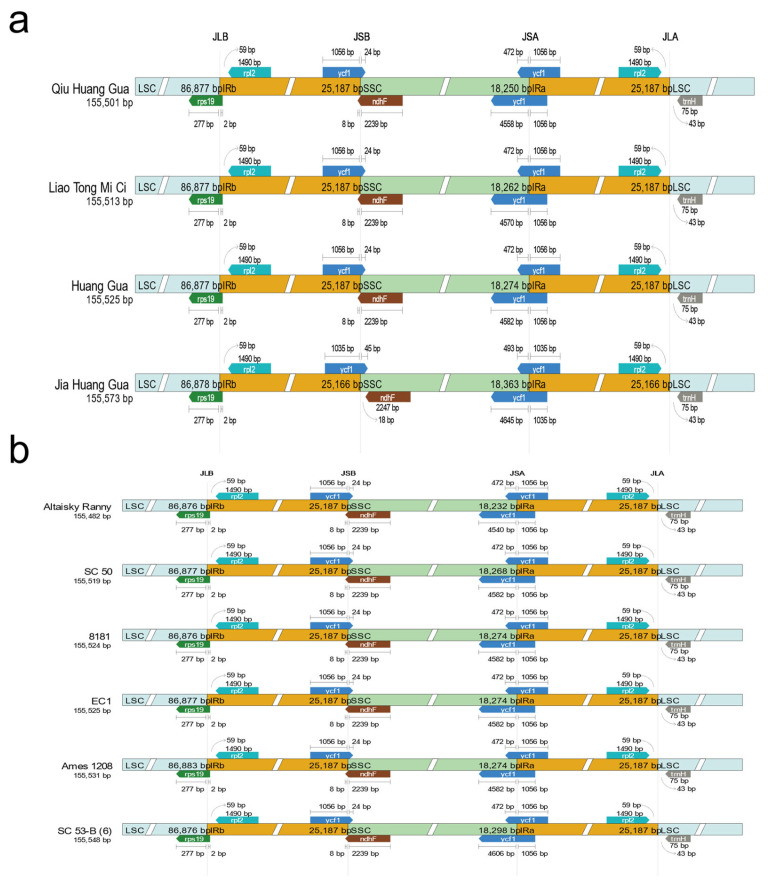

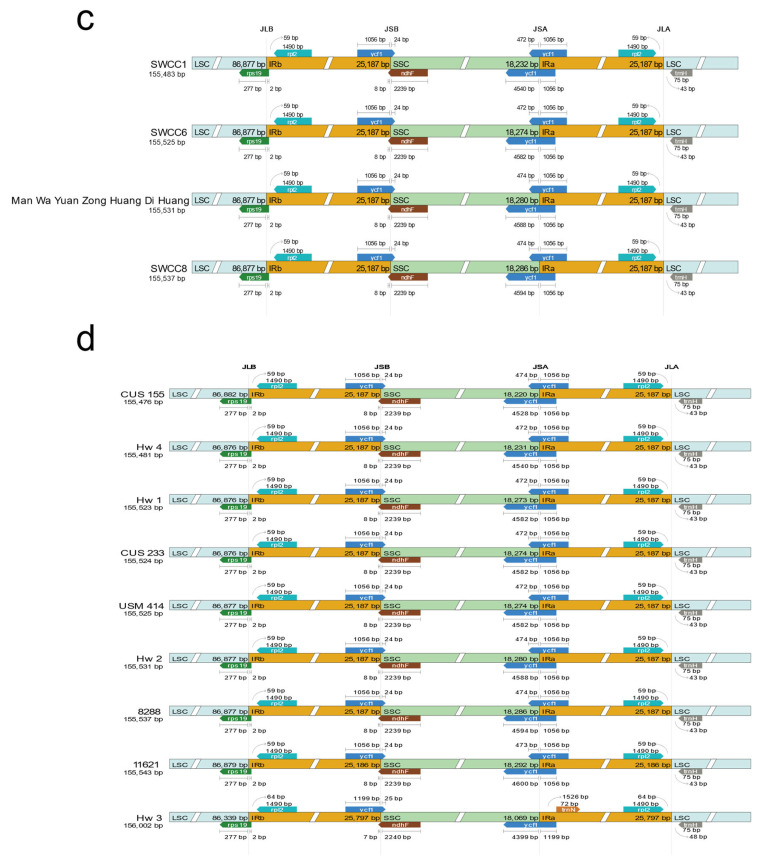
Comparison of the borders of the LSC, SSC, and IR regions in East Asian type (**a**), Eurasian-type (**b**), Xishuangbanna-type (**c**), and Indian-type (**d**) cucumbers.

Chloroplast DNA is usually used to explore genetic diversity within species, and expansion or narrowing of the IR region in the chloroplast genome can lead to size variation at the LSC/IRB/SSC/IRA boundary. In this study, we performed a comparative analysis of cucumber chloroplast genomes. When comparing the chloroplast genomes of different cucumber ecotypes, we found that ycf1 was the only difference in the IRa-SSC (JSA) (Figure S2). When comparing the chloroplast genomes of the East Asian-type cucumbers (Figure 2a), we found that the four boundaries were relatively conserved, differing only in ycf1 for the IRa-SSC (JSA) and IRb-SSC (JSB) boundaries. The results of Eurasian-type (Figure 2b) and Xishuangbanna-type (Figure 2c) cucumber chloroplast genome comparative analyses found that only the JSB boundary differs between the two. However, when performing chloroplast genome comparative analysis of Indian-type (Figure 2d) cucumbers, it is found that they differed in the four boundaries of JSA, JSB, IRa-LSC (JLA), and IRb-LSC (JLB). These results also indicate that there is expansion and contraction of chloroplast DNA boundary regions between different species of the same type studied, most notably in the Indian-type cucumbers.

**Figure 3:**
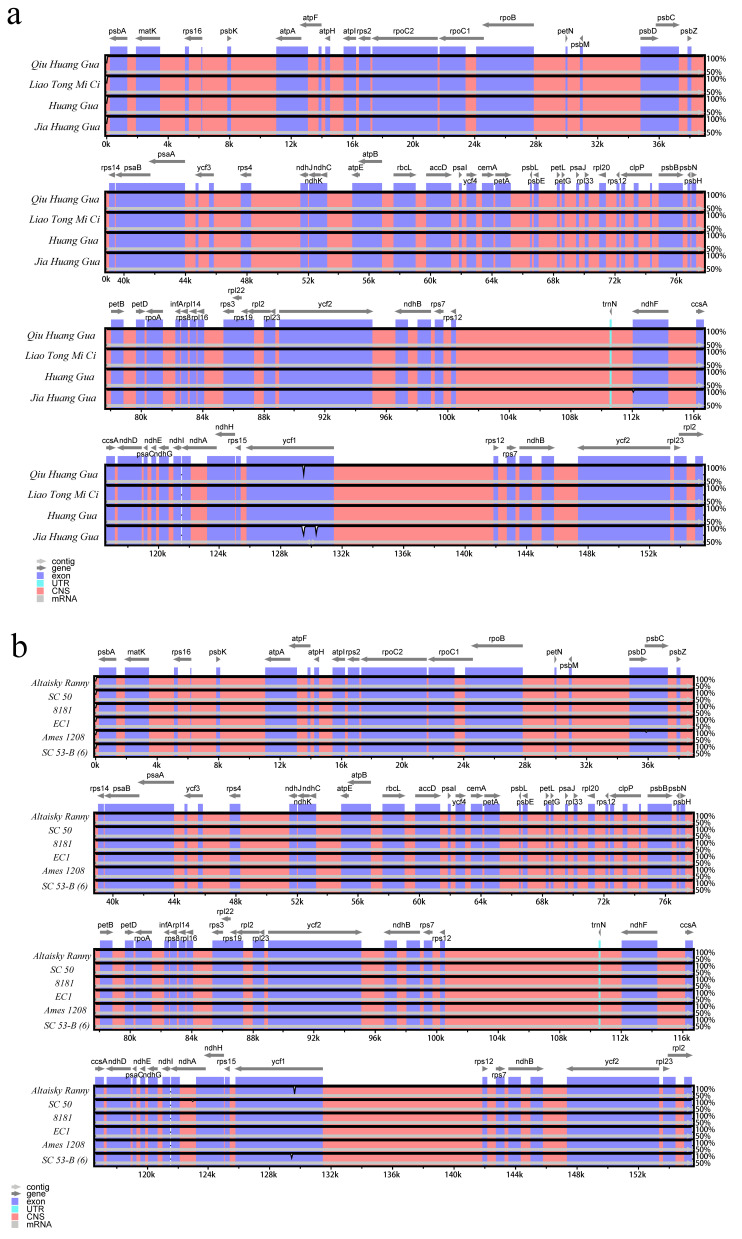

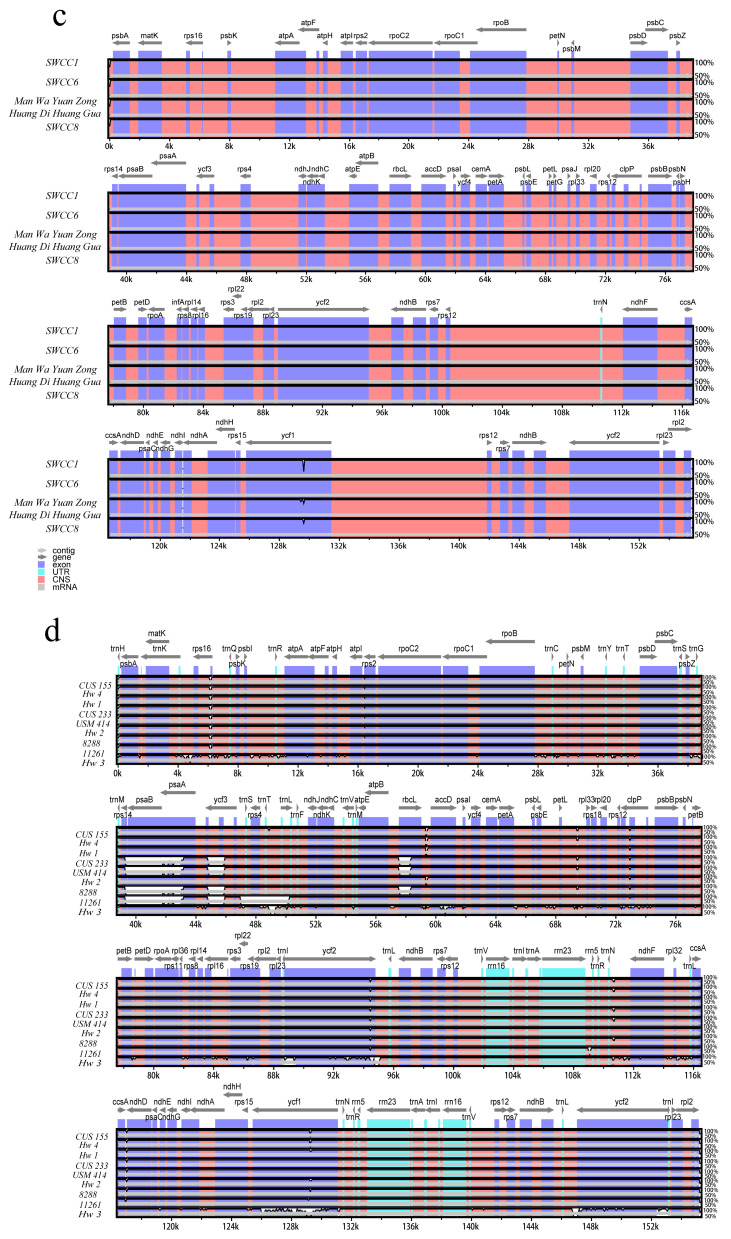
Comparison of chloroplast genomes of East Asian-type (**a**), Eurasian-type (**b**), Xishuangbanna-type (**c**), and Indian-type (**d**) cucumbers by mVISTA software.

To further analyze the potential divergence of these genomic sequences, we used mVISTA to calculate sequence identity. The GY14 (DQ865975.1) and *Cucumis sativus* var. hardwickii (KT852702.1) chloroplast genomes were downloaded from NCBI and used for comparative analysis. The results found that the LSC region was more diverse than the SSC and IR regions in all ecotypes. In the East Asian-type (Figure 3a) and Xishuangbanna-type (Figure 3c) cucumbers, we found relatively high levels of variation in the ycf1 region, such as Qiu Huang Gua and Jia Huang Gua. In the Eurasian-type cucumbers, we found that Ames1208 (Figure 3b) has higher levels of variation in the trnS-trnM, psaB, atpB-ycf3, and ycf1 regions than in other materials. Similarly, it is found that psaB, atpB-ycf3, rbcL, rbcL-accD, clpP, trnN-ndhF, and ycf1 regions have high levels of variation in the Indian-type (Figure 3d) cucumbers. These results are consistent with the above boundary results.

### 2.3. Phylogenetic Analysis and Species Delimitation

**Figure 4:**
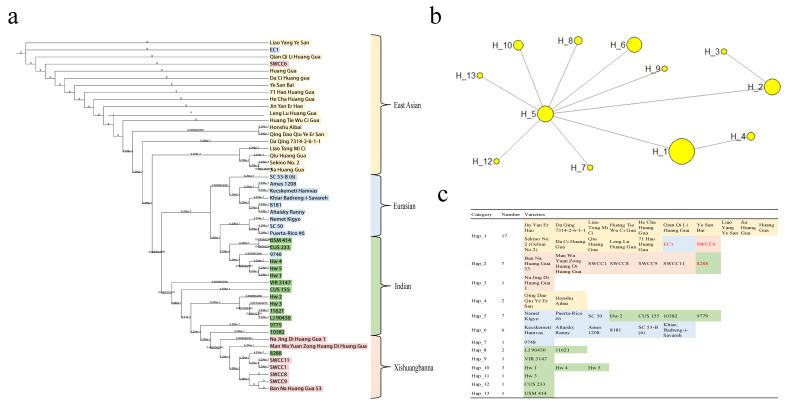
Phylogenetic analysis and species delimitation in cucumbers. (**a**) The ML phylogenetic tree based on complete chloroplast genomes. Haplotype analysis (**b**) and classification (**c**) of 50 cucumber chloroplast genomes.

An ML tree was constructed based on the whole chloroplast genome to study the phylogenetic relationships of cucumbers. The tree shows that the 50 cucumber germplasm resources were divided into 3 main branches (Figure 4a): East Asian, Eurasian + Indian, and Xishuangbanna + Indian. The phylogenetic analysis found that Indian-type cucumbers are more closely related to Xishuangbanna-type cucumbers. Interestingly, we found three species excluded from their original ecotype classification: EC1, SWCC6, and 8288. This result might be caused by the extensive mutual introduction and cross-fertilization of different ecotypes of cucumber. Meanwhile, haplotype analysis was performed using 50 cucumber chloroplast genomes. The results classified 50 cucumbers into 13 categories (Figure 4b). Among them, East Asian and Xishuangbanna were divided into 2 groups, and Eurasian and Indian-type were divided into 3 and 7 groups, respectively. The results of EC1, SWCC6, and 8288 divisions were consistent with the results of evolutionary tree analysis (Figure 4c), proving the accuracy and robustness of the results obtained in this study. On the other hand, it turned out that the Indian type has more haplotypic material compared to other ecotypes, demonstrating its rich genetic diversity.

### 2.4. Genetic Variation and Structure Analysis

**Figure 5:**
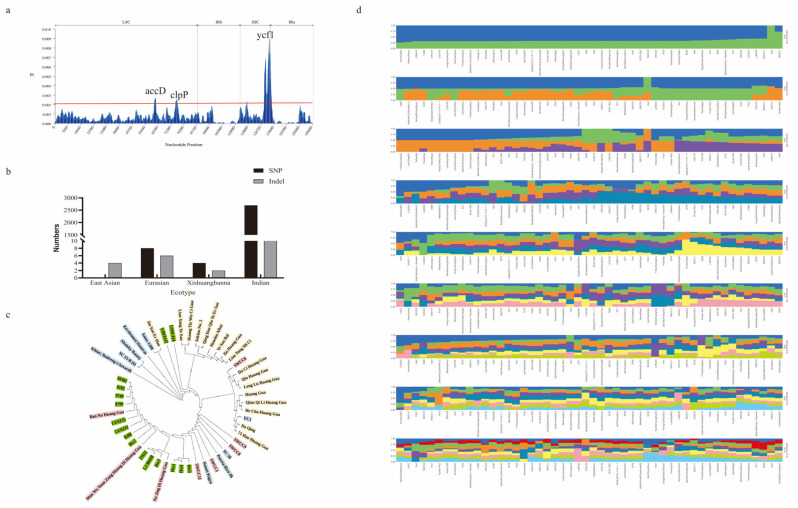
Genetic variation and structure analysis of 50 cucumber chloroplast genomes. (**a**) Statistics of nucleotide diversity (Pi) in 50 cucumber chloroplast genomes with parameters of a window length of 1000 bp and a step size of 100 bp. (**b**) The variations in 50 cucumber chloroplast genomes. The phylogenetic tree (**c**) and population structure analyses (**d**) are based on SNP.

To investigate the genetic diversity of cucumber chloroplast genomes, we analyzed the nucleotide diversity (Pi) values and variants of 50 cucumber chloroplast genomes. Highly variable and significantly higher Pi values > 0.002 were found in ycf1, accD, clpP. Among them, the ycf1 region had the highest divergence value, which was 0.01. The gene *ycf1* harbored the most variants, which corresponded to the results of the comparative genome analysis. Variants in cucumber chloroplast DNA were detected in each ecotype (Figure 5b). Among the East Asian, Eurasian, and Xishuangbanna types, 4 (4 Indels), 14 (8 SNPs + 6 Indels), and 6 (4 SNPs + 2 Indels) variants were found, respectively. Unlike the aforementioned types, a large number of genetic variants were found in the Indian type, including 2695 SNPs and 376 Indels. These results demonstrate that Indian cucumbers have more genetic variation compared to other cucumbers.

Phylogenetic tree and population structure analyses were performed by extracting SNPs. The phylogenetic tree analysis showed that four major groups were clustered (Figure 5c), which is generally in agreement with the chloroplast genome phylogenetic tree results. However, Jin Yan Er Hao did not cluster with the East Asian cucumber, which may be caused by differences in mutation rates between coding and non-coding regions of the chloroplast genome. Population structures were analyzed, with the K value ranging from 2 to 10 (Figure 5d). The cross-validation (CV) error was the lowest with K = 9 (Figure S3). Taken together, the findings suggested that the cucumber population could be divided into nine groups.

### 2.5. Response of Plastid Genes to Temperature Stress in Cucumber

**Figure 6:**
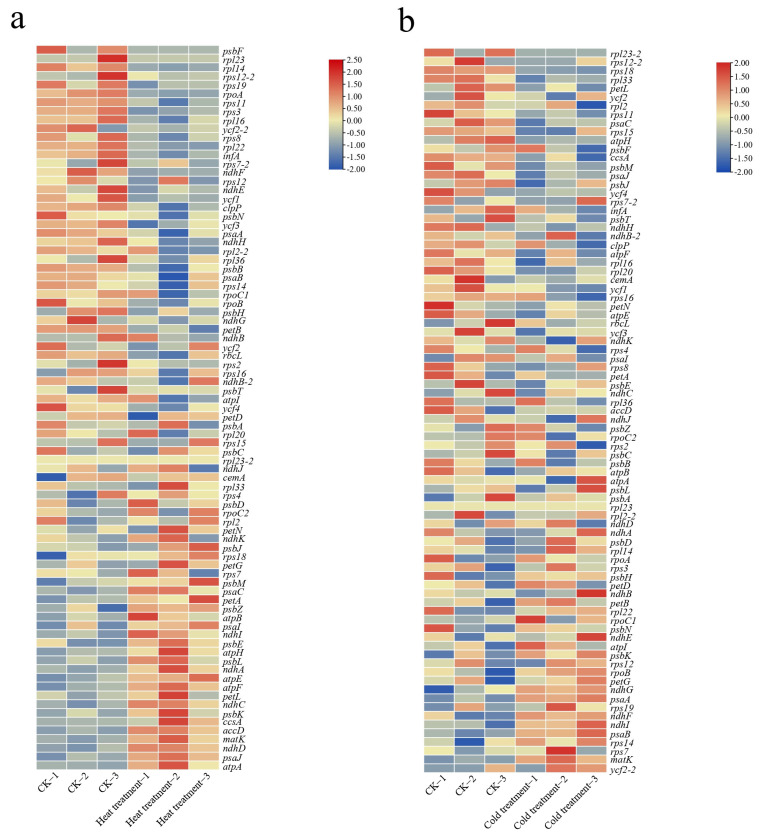
Heatmap of plastid gene expression under temperature stress in the cucumber. (**a**) Heatmap of plastid gene expression under heat stress and at normal temperature (CK). (**b**) Heatmap of plastid gene expression under cold stress and at normal temperature (CK).

Chloroplasts are thought to be involved in temperature sensing and adaptive regulation, which are key components of the response to temperature stimuli. We use transcriptome data to mine how temperature stresses affect the development of plant plastids. After the high-temperature treatment, we found that a large number of chloroplast genes were down-regulated in expression (Figure 6a, Table S4). Among them, genes related to ribosome metabolism in photosynthesis were significantly inhibited, such as *rps3*, *rps8*, *rps9*, and *rpl14*, among others. This indicates that ribosomal metabolism is hindered under high-temperature conditions and is an important factor affecting chloroplast development and photosynthesis in plants. Interestingly, we found that the expression of nine genes were up-regulated, including *accD* and *matK*, both of which are closely related to lipid synthesis. It is hypothesized that plants under high-temperature stress resist adversity stress by regulating lipid metabolism. After low-temperature treatment, we found significant inhibition of genes related to ribosome metabolism (Figure 6b, Table S5), such as *rps18* and *rpl33*. These results suggest that ribosome metabolism is the single most important factor affecting plant chloroplast development and photosynthesis under temperature adversity stress. Similarly, we found that *matK* was significantly up-regulated at low temperatures, demonstrating that chloroplast resistance to temperature stress through the regulation of lipid metabolism is an important pathway for plant stress tolerance.

**Figure 7:**
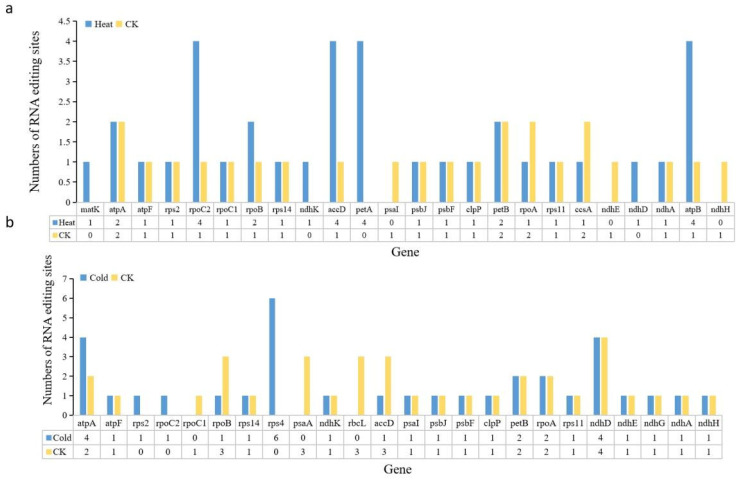
Analysis of RNA editing efficiency in plastid genes under temperature stress in cucumber. (**a**) Analysis of RNA editing efficiency of heat stress and normal temperature (CK). (**b**) Analysis of RNA editing efficiency of cold stress and normal temperature (CK).

RNA editing plays an important role in plant growth, development, and evolutionary adaptation, and environmental stress can affect the efficiency of RNA editing. We used transcriptome data to analyze the RNA editing efficiency of plant plastid genes under temperature stress. After high-temperature treatment, we found RNA editing events in 21 genes and identified 36 editing sites (Figure 7a, Table S6), which far exceeds the 24 editing sites detected at normal temperature (Table S7). Of these, a total of 25 were C-to-U, accounting for 69.44% of RNA editing events, which is lower than the C-to-U editing efficiency of 83.33% at normal temperature. Compared with normal temperature, the RNA editing efficiency of *matK*, *accD*, *atpB*, *rpoC2*, and *petA* was significantly improved under high-temperature conditions. Among them, we found that the increased efficiency of *accD* and *matK* RNA editing may promote the expression of accD and matK in response to high-temperature stress. After low-temperature treatment, RNA editing events occurred in 21 genes, and 34 editing sites were identified (Figure 7b, Table S8). Of those 34 editing events, 29 were C-to-U, accounting for 85.29% of RNA editing events, which were not significantly different from the 27 C-to-U editing events detected at normal temperature (Table S9). Compared with normal temperature, we found that the RNA editing efficiency of *atpA*, *rps2* and *rps4* was significantly improved, while the RNA editing efficiency of *rpoB*, *psaA*, *rbcL* and *accD* was significantly reduced in low-temperature conditions. These results suggested that low temperature leads to a decrease in editing efficiency of photosynthetic genes (*rpoB*, *psaA*, and *rbcL*), which may further affect plant photosynthesis.

## 3. Discussion

In this study, 50 cucumber chloroplast genomes were assembled and annotated. This is the first report on the cucumber chloroplast pan-genome, which is important for exploring genetic diversity and chloroplast genome evolution in cucumbers. The 50 cucumber chloroplast genomes showed high similarity in genome structure, GC content, and gene composition with previously reported cucumber chloroplast genomes [9,11]. However, compared to other cucumbers, the chloroplast genomes of Indian cucumbers have more variations, such as genome size. Comparative analysis of chloroplast genome size in Indian cucumbers revealed that the differences were mainly in the LSC and SSC regions, which is consistent with the results of *Eriocaulon* [33]. Expansion and contraction of the chloroplast genome is a common evolutionary phenomenon in plants, which can be altered by IR contraction and expansion [34]. Analysis of 50 cucumber germplasm IRB/SSC/IRA/LSC boundaries revealed that the IR region is conserved and most of the substitutions occur in the SSC and LSC regions. This is similar to findings in the plastid genomes of species such as *Yam* [35], *Nicotiana* [36], and *Mukdenia* [37].

The chloroplast genome regions (atpF-H, matK, and rbcL) have been used as candidate markers for DNA barcoding in plants to facilitate rapid identification of species [38,39]. Based on the results of mVISTA, nucleotide diversity, and comparative chloroplast genome analyses, 8 highly variable regions were identified in 50 cucumbers, including 3 intergenic regions (rbcL-accD, atpB-ycf3, and ndhF-trnN) and 5 gene regions (rbcL, psaB, clpP, accD, and ycf1). Previous studies have shown that ycf1 shows large variations in different species and is used as a chloroplast DNA barcode to identify different species [40]. In this study, ycf1 was highly variable between different cucumbers (Figure S4), whose coding sequences can be used to distinguish different cucumber ecotype germplasm resources.

The chloroplast genome has been used as an effective marker for studying species diversity in many plants [21]. Genetic diversity of cucumber germplasm was detected based on 37 SSR markers in non-coding regions of the chloroplast genome [11]. The conduct of chloroplast pangenome studies could provide a new tool to explore the genetic diversity of cucumber germplasms. Based on phylogenetic tree results, the 50 cucumber germplasms were divided into 4 major branches: East Asian, Eurasian, Xishuangbanna, and Indian, which are the same as the previous phylogenetic relationship of the nuclear genome [3]. However, some varieties were not well-differentiated at the chloroplast genome level, indicating that the chloroplast genome of cucumber is conserved within the species. Among these, Indian wild cucumbers were more distantly related to the East Asian cultivated cucumbers, which could be the result of artificial domestication selection [4]. In terms of phylogenetic relationships, the Indian cucumbers and the Xishuangbanna cucumbers are closely related, likely due to their close geographical location and similar ecological environment [41]. Interestingly, we found that the three varieties—EC1, SWCC6, and 8288—did not cluster together with their original ecotype classification. The conflict of phylogenetic trees between chloroplast genomes and nuclear genes is also common in angiosperms [42,43]. This result may be caused by extensive mutual introgression and cross-fertilization of different cucumber ecotypes, which is consistent with citrus results [43].

Genetic diversity and population structure of cucumber varieties have been studied using resequencing data from 115 cucumbers. The results of phylogenetic evolution in the nuclear genome classified 115 cucumbers into 4 groups (East Asian, Eurasian, Xishuangbanna, and Indian), consistent with the chloroplast genome phylogenetic findings [3]. Molecular markers such as SSR and SNP can be used for variety identification and genetic evolution studies [44,45,46,47,48]. In this study, we extracted SNPs and performed a genetic structure analysis on resequencing data from 50 cucumbers. The haplotype and population genetic structure results found that the Indian cucumber is rich in genetic diversity and has more haplotypes, which is the same as the previous resequencing results [6]. However, the chloroplast genomes of different germplasms of the same ecotype are indistinguishable, such as “Huang Gua and Da Ci Huang Gua” in the East Asian type, likely due to characteristics such as a highly conserved chloroplast genome and little intraspecific genetic variation within species.

Chloroplasts play a central role in the perception and integration of temperature stresses in plants [49]. Significant down-regulation of ribosomal protein genes in the chloroplast genome of cucumber was also identified in this study through the analysis of transcriptome data from high- and low-temperature stresses. Plastid ribosomal proteins are an important component of the protein synthesis machinery and have multiple roles in plant growth and development [50]. Previous studies have found that overexpression of the *rps5* gene improves plant cold tolerance in *Arabidopsis* [51], while reduced expression of *rps1* causes a significant reduction in plant heat tolerance [52]. RNA editing regulates the expression of organelle genes in plants in response to different environmental stress [53]. Our study found that *accD* exhibited significantly higher expression in high- and low-temperature treatments, which may be a defense measure of plant chloroplasts in response to temperature stimuli. Meanwhile, *accD* showed a remarkable increase in RNA editing efficiency under high-temperature conditions. Fatty acid biosynthesis is initiated in the chloroplast under the control of the *accD* gene [54]. Improving the editing efficiency of *accD* in plants significantly enhanced heat tolerance in *Arabidopsis* [55], implying that tolerance in plants such as cucumber could be improved through plastid transgenesis. C-to-U RNA editing events occur frequently in organelle genes of plants. Previous studies have found that the RNA editing efficiency of C-to-U in the *Arabidopsis* organelle genomes is reduced in response to heat stress, which is associated with stalling of ribosomal genes [56]. Similarly, it was found that the editing efficiency of C-to-U in the chloroplast genome of cucumber was reduced after heat treatment, which implied that the reduced rate of C-to-U RNA editing might be involved in regulating the stress response of plants.

## 4. Materials and Methods

### 4.1. Plant Material and Sequencing

The 121 cucumber germplasm resources used in this study were derived from 115-core cucumber germplasm collections and 6-core germplasms from our lab (Table S10). Among the germplasm collections, the resequenced data of 115-core germplasm resources were downloaded from the SRA database (SRA056480) on the NCBI website. The 6-core germplasms were grown in a greenhouse at Baima Teaching and Research Base of Nanjing Agricultural University. Total genomic DNA was extracted from fresh leaves using a modified CTAB method [57]. DNA concentration and quality were measured using a NanoDrop 2000 spectrophotometer (Thermo Scientific, Carlsbad, CA, USA). The qualified DNA samples were sequenced on the Illumina NovaSeq 6000 platform (Illumina Inc., San Diego, CA, USA).

### 4.2. Assembly and Annotation of Chloroplast Genomes

The fastq-dump in the SRA toolkit (https://ftp-trace.ncbi.nlm.nih.gov/sra/sdk/2.8.0/, accessed on 3 April 2023) was used to convert the SRA data from NCBI to the fastq file. The raw reads were trimmed and quality-controlled by Trimmomatic [58] to harvest the clean reads, which were used for the chloroplast genome assembly. The chloroplast genome was assembled by GetOrganelle software to obtain chloroplast circular DNA molecules [59]. The complete chloroplast genome sequences were compared with the reference genome GY14 (DQ865975.1) using TBtools software [60] to verify the accuracy of assembly. Chloroplast genes were annotated by module GeSeq [61] and manually corrected. Organellar Genome DRAW was used to draw the chloroplast map.

### 4.3. Comparative Analysis of Chloroplast Genomes

The IRscope [62] was used to detect LSC/IRB/SSC/IRA boundaries between the chloroplast genome sequences of different cucumbers for comparative analysis. The chloroplast genomes of different cucumbers were compared using the mVISTA program [63]. The annotation of GY14 (DQ865975.1) and *Cucumis sativus* var. hardwickii (KT852702.1) was used as a reference in the Shuffle-LAGAN model, where *Cucumis sativus* var. hardwickii (KT852702.1) was used for comparison with Indian-type cucumbers.

### 4.4. Phylogenetic Tree and Haplotype Analysis

This phylogenetic tree analysis was constructed based on the complete chloroplast genome sequences of 50 cucumber germplasms. All sequences were aligned using MAFFT software with default settings [64]. The phylogenetic tree was created using IQ-TREE 2 [65] by selecting the maximum likelihood (ML) technique and 1000 bootstrap replications through Ultrafast bootstrap parameters. It was visualized and modified by iTOL (interactive tree of life). Nucleic acid diversity and haplotype analyses of the chloroplast genome were performed with DnaSP 6 software [66]. Haplotype network diagrams were drawn using network software (https://www.fluxus-engineering.com/sharenet.htm, accessed on 3 April 2023).

### 4.5. Variants Calling

Clean reads were aligned to the reference chloroplast genome by BWA to obtain comparison files [67]. Used SAMtools and BCFtools to call the variants from the comparison files [68,69]. SNPs and InDels were filtered using VCFtools [70], setting parameters for deletion rates below 50% and minor allele frequencies above 0.05.

### 4.6. Phylogenetic Tree and Population Genetic Structure Based on Variants

Plink [71] was used to convert the vcf format into the phylip format, which was used for the phylogenetic tree analysis. FastTree [72] was selected to construct the phylogenetic tree by the ML method. CV errors were assessed using ADMIXTURE [73], setting parameters from K = 2 to K = 10. The visualization was performed by the R package (bar graph).

### 4.7. RNA-Seq Analysis

RNA-Seq data were downloaded from the SRA database (SRP305598 and SRP262962) on the NCBI website. The fastq-dump in the SRA toolkit (https://ftp-trace.ncbi.nlm.nih.gov/sra/sdk/2.8.0/, accessed on 3 April 2023) was used to convert the data to the fastq file. The raw reads’ quality was trimmed by Trimmomatic software [58]. The quality-controlled reads were aligned to the chloroplast genome using Hisat2 [74]. Mapped reads were counted using featureCounts [75]. The comparative analysis of gene expression levels between samples was performed using DEseq2 [76]. Fragments per kilobase million (FPKM) were calculated, and differentially expressed genes (DEGs) were defined as those with a fold change (FC) > 2 between samples and a *p*-value < 0.05.

### 4.8. Analysis of RNA Editing Efficiency

RNA-Seq data were downloaded from the SRA database (SRP305598 and SRP262962) on the NCBI website. The fastq-dump in the SRA toolkit (https://ftp-trace.ncbi.nlm.nih.gov/sra/sdk/2.8.0/, accessed on 3 April 2023) was used to convert the data to the fastq file. The raw reads’ quality was trimmed by Trimmomatic software [58]. The quality-controlled reads were aligned to the chloroplast genome using BWA [67]. Use GATK [77] to mine variation information such as SNP and Indel. REDO [78] was used to detect RNA editing sites in the chloroplast genome in the variant calling files. If there were 2 or more RNA editing site events in 3 replicate samples, RNA editing was considered to have occurred.

## 5. Conclusions

In this study, we assembled and compared 50 cucumber chloroplast genomes, and found 8 highly variable regions that can be used as potential sources of molecular markers for species identification. The haplotype and population genetic structure results revealed that the Indian-type cucumbers have more genetic variation compared to other cucumbers, which means that many genetic resources are remaining to be explored in the Indian-type cucumbers. Transcriptome results showed that *accD*, *matK*, and ribosomal protein genes in the chloroplast genome expression were disrupted under temperature stimulation. Taken together, our results provide useful information on genetic variation and adaptation to temperature stress in the chloroplast genome.

## Data Availability

All relevant data analyzed during this study are included in this article and additional files. The SRR numbers of all resequencing and transcriptome data analyzed during this study are in Supplementary File S1. The 50 chloroplast genome sequences were released to GenBank and their accession numbers are available in Supplementary File S1.

## Supplementary Materials

The following supporting information can be downloaded at: https://www.mdpi.com/article/10.3390/ijms24108943/s1.
