# Supplementary material for: Chloroplast Pan-Genomes and Comparative Transcriptomics Reveal Genetic Variation and Temperature Adaptation in the Cucumber
Source: Int J Mol Sci. 2023 May 18;24(10):8943. doi: 10.3390/ijms24108943 (PMC10219501; doi:10.3390/ijms24108943)
Supplement: Supplementary file 1 [file ijms-24-08943-s001.zip › ijms-2355541-supplementary.pdf]

Chloroplast Pan-Genomes and Comparative Transcriptomics Reveal Genetic Variation and Temperature Adaptation in the Cucumber

Lei Xia [1], Han Wang [1], Xiaokun Zhao [1], Hesbon Ochieng Obel [1], Xiaqing Yu [1], Qunfeng Lou [1], Jinfeng Chen [1,*], and Chunyan Cheng [1,*]

[1]National Key Laboratory of Crop Genetics & Germplasm Enhancement and Utilization, College of Horticulture, Nanjing Agricultural University, Nanjing 210095, China

[*]Correspondence: jfchen@njau.edu.cn; Tel.: +86-025-8439-6370

[*]Correspondence: chunyancheng@njau.edu.cn; Tel.: +86-025-8439-6370

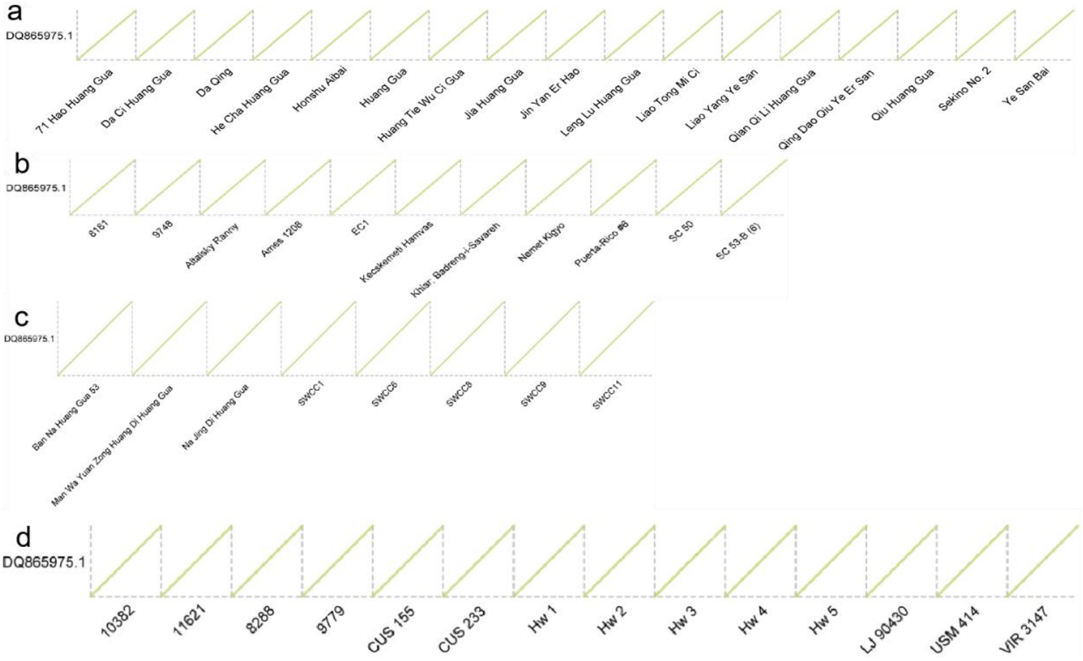

Figure S1. Co-linearity analysis of our assembled chloroplast genome with "GY14" reference chloroplast genome (DQ865975.1).

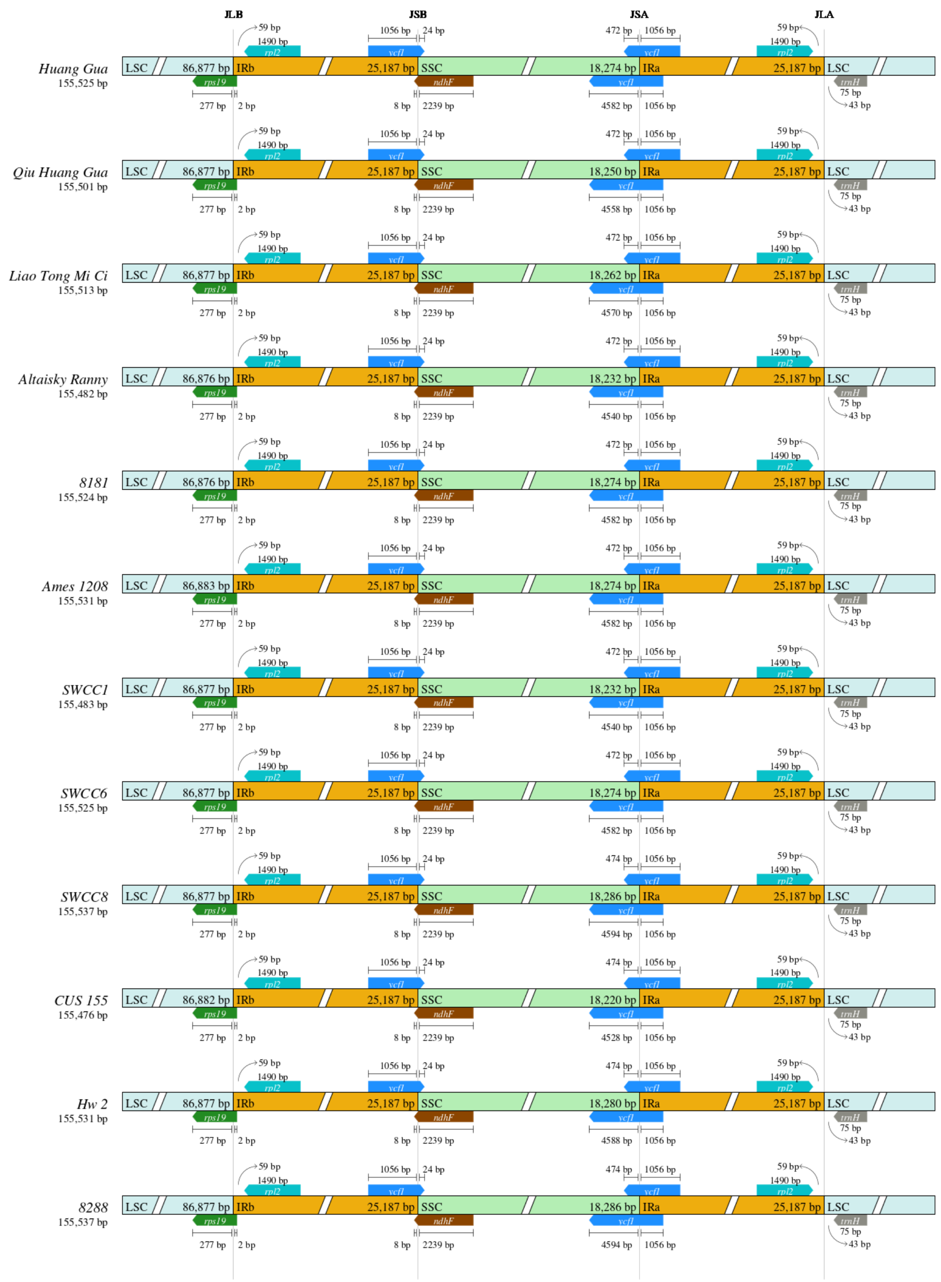

Figure S2. Comparison of the borders of the LSC, SSC, and IR regions in different ecotype cucumbers.

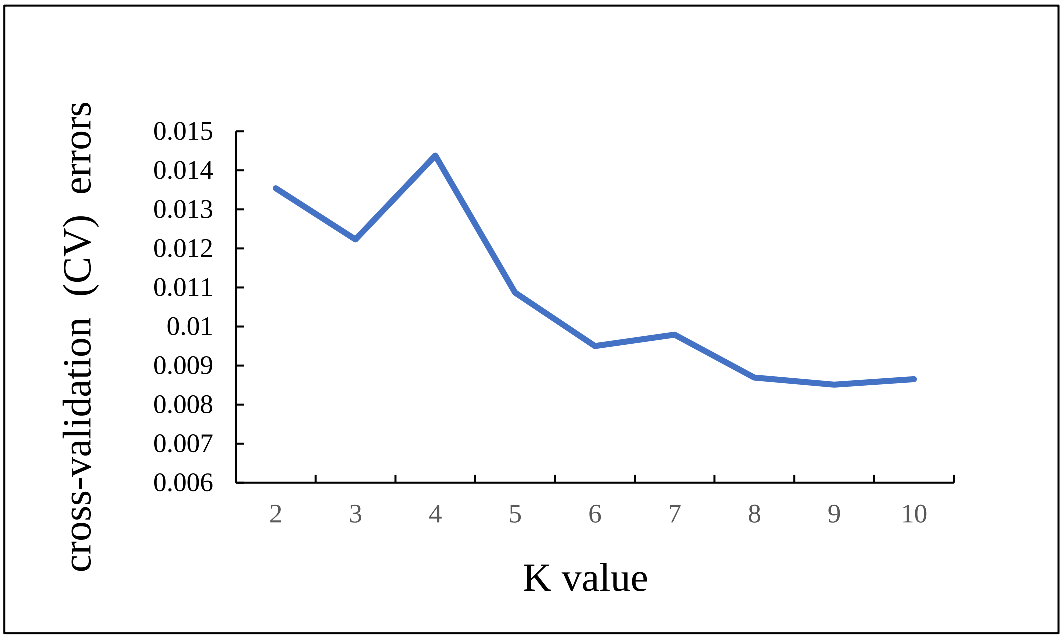

Figure S3. The CV error with different K value.

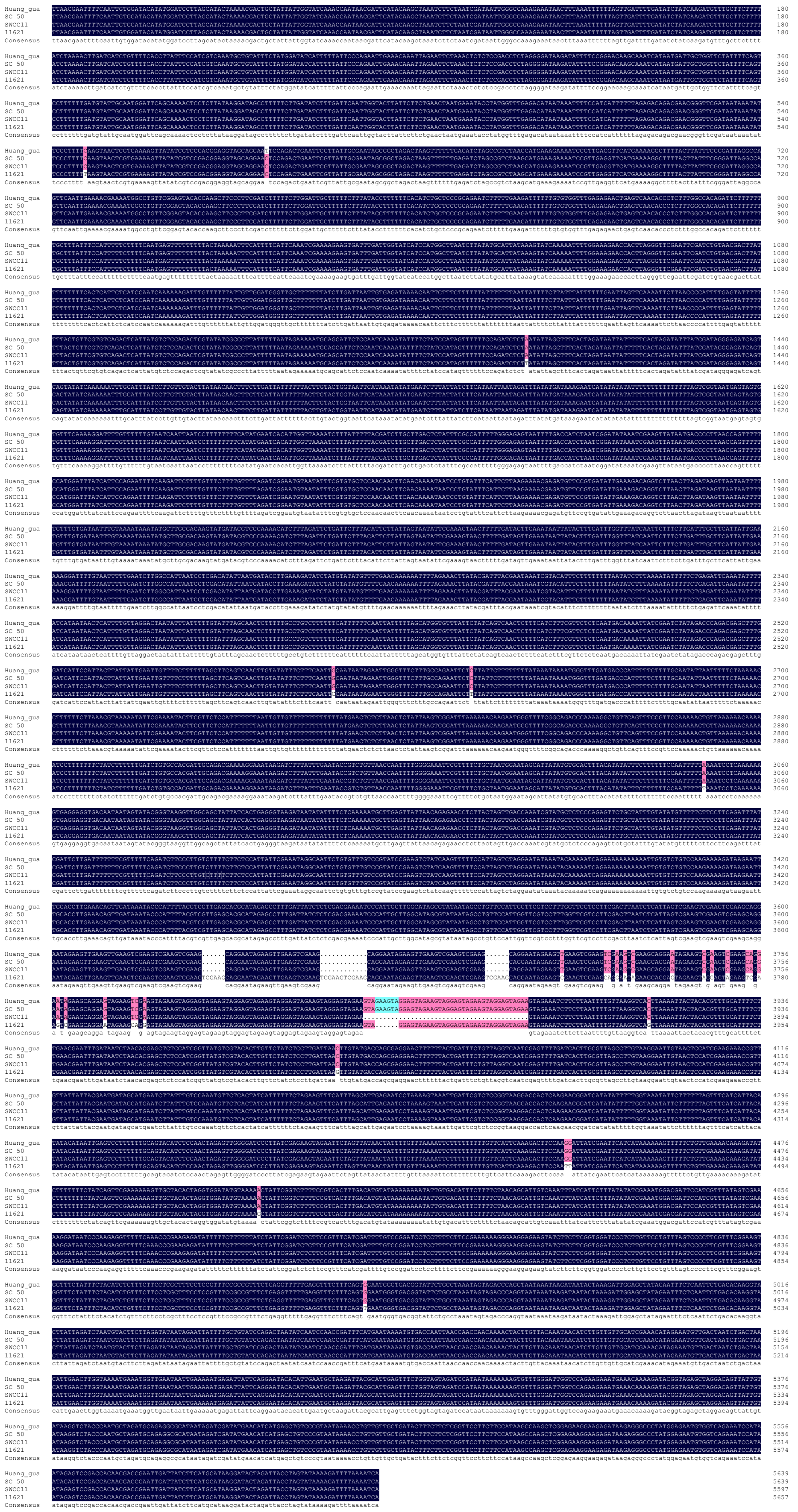

Figure S4. The differences in ycf1 coding sequence of Huang gua (East Asian), SC 50 (Eurasian),

SWCC11 (Xishuangbanna) and 11621 (Indian).

Table S10. The SRR number, Genebank accession number and corresponding ecological type of cucumber varieties used in this study.

Samples	SRRNumebr	Ecotype	Genebank accession number
DaQing7314-2-6-1-1	SRR543220	EastAsian	OQ675040
LiaoTongMiCi	SRR543221	EastAsian	OQ675041
HuangTieWuCiGua	SRR543222	EastAsian	OQ675042
HeChaHuangGua	SRR543223	EastAsian	OQ675043
QianQiLiHuangGua	SRR543224	EastAsian	OQ675044
YeSanBai	SRR543225	EastAsian	OQ675045
LiaoYangYeSan	SRR543226	EastAsian	OQ675046
JiaHuangGua	SRR543229	EastAsian	OQ675047
HuangGua	SRR543230	EastAsian	OQ675048
QingDaoQiuYeErSan	SRR543231	EastAsian	OQ675049
HonshuAibai	SRR543238	EastAsian	OQ675050
SekinoNo.2(OchiaiNo.2)	SRR543239	EastAsian	OQ675051
DaCiHuangGua	SRR543242	EastAsian	OQ675052
QiuHuangGua	SRR543243	EastAsian	OQ675053
LengLuHuangGua	SRR543244	EastAsian	OQ675054
71HaoHuangGua	SRR543245	EastAsian	OQ675055
JinYanErHao	SRR543267	EastAsian	OQ675061
NemetKigyo	SRR543254	Eurasian	OQ675056
KecskemetiHamvas	SRR543255	Eurasian	OQ675057
AltaiskyRanny(AltaiskiRannii166)	SRR543260	Eurasian	OQ675058
Ames1208	SRR543262	Eurasian	OQ675059
8181	SRR543266	Eurasian	OQ675060
Puerta-Rico#6	SRR543268	Eurasian	OQ675062
SC53-B(6)	SRR543270	Eurasian	OQ675063
SC50	SRR543272	Eurasian	OQ675064
Khiar;Badreng-i-Savareh	SRR543277	Eurasian	OQ675065
9748	SRR543280	Eurasian	OQ675066
EC1	SRR11300861	Eurasian	OQ675036
BanNaHuangGua53	SRR543310	Xishuangbanna	OQ675037
ManWaYuanZongHuangDiHuangGua	SRR543311	Xishuangbanna	OQ675038
NaJingDiHuangGua1	SRR543313	Xishuangbanna	OQ675039
SWCC1	SRR23913619	Xishuangbanna	OQ675081
SWCC6	SRR23913618	Xishuangbanna	OQ675082
SWCC8	SRR23913617	Xishuangbanna	OQ675083
SWCC9	SRR23913616	Xishuangbanna	OQ675084
SWCC11	SRR23913615	Xishuangbanna	OQ675085
LJ90430	SRR543203	Indian	OQ675067
11621	SRR543204	Indian	OQ675068

VIR3147	SRR543207	Indian	OQ675069
Hw1	SRR543208	Indian	OQ675070
Hw2	SRR543209	Indian	OQ675071
Hw3	SRR543210	Indian	OQ675072
Hw4	SRR543211	Indian	OQ675073
Hw5	SRR543212	Indian	OQ675074
CUS233	SRR543213	Indian	OQ675075
CUS155	SRR543214	Indian	OQ675076
USM414	SRR543288	Indian	OQ675077
10382	SRR543289	Indian	OQ675078
9779	SRR543295	Indian	OQ675079
8288	SRR543298	Indian	OQ675080
